# Iturin A Potentiates Differentiation of Intestinal Epithelial Defense Cells by Modulating Keap1/Nrf2 Signaling to Mitigate Oxidative Damage Induced by Heat-Stable Enterotoxin B

**DOI:** 10.3390/antiox14040478

**Published:** 2025-04-16

**Authors:** Geng-Xiu Zan, Hao-Zhan Qu, Xin-Yang Li, Qi-Liang Peng, Xiao-Fan Wang, Run-Sheng Li, Yu-Guang Zhao, Hui-Chao Yan, Jia-Yi Zhou, Xiu-Qi Wang

**Affiliations:** 1 State Key Laboratory of Swine and Poultry Breeding Industry, College of Animal Science, South China Agricultural University, National Engineering Research Center for Breeding Swine Industry, Guangdong Laboratory for Lingnan Modern Agriculture, Guangdong Provincial Key Laboratory of Animal Nutrition Control, Guangzhou 510642, China; zangengxiu@stu.scau.edu.cn (G.-X.Z.); 1824181951@stu.scau.edu.cn (H.-Z.Q.); lixinyang@stu.scau.edu.cn (X.-Y.L.); 20213139086@stu.scau.edu.cn (Q.-L.P.); xxw033@scau.edu.cn (X.-F.W.); yanhc@scau.edu.cn (H.-C.Y.); jyzhou@scau.edu.cn (J.-Y.Z.); 2 Department of Infectious Diseases and Public Health, City University of Hong Kong, Hong Kong 518057, China; runsheng.li@cityu.edu.hk; 3 Division of Structural Biology, Nuffield Department of Medicine, Oxford University, Oxford OX3 7BN, UK; yuguang.zhao@strubi.ox.ac.uk

**Keywords:** mouse, STb, iturin A, intestinal stem cell, Keap1/Nrf2 signaling

## Abstract

Intestinal stem cells (ISCs) maintain epithelial renewal through their proliferation and differentiation capabilities, responding to various intestinal insults. However, the impact of iturin A, a natural antimicrobial peptide, on ISC viability and its potential to mitigate heat-stable enterotoxin b (STb)-induced intestinal damage remains unclear. Our recent study demonstrated that oral administration of iturin A enhances tight junction protein expression, accelerates crypt-villus regeneration, and restores epithelial barrier integrity in STb-exposed mice. Furthermore, iturin A promotes ISC proliferation and differentiation, significantly increasing the numbers of goblet and Paneth cells in the jejunum following STb exposure. Notably, iturin A regulates intestinal homeostasis by scavenging reactive oxygen species (ROS), while elevating total antioxidant capacity (T-AOC), superoxide dismutase (SOD), and glutathione peroxidase (GSH-PX) levels in both serum and jejunal mucosa. Mechanistically, iturin A facilitates nuclear factor-erythroid 2- related factor 2 (Nrf2) release by disrupting Kelch-like ECH-associated protein 1 (Keap1), leading to the upregulation of the antioxidant enzyme glutathione peroxidase 4 (GPX4). In conclusion, our findings indicate that iturin A alleviates oxidative stress induced by STb through modulation of the Keap1/Nrf2 pathway and promotes ISC differentiation into goblet and Paneth cells, thereby enhancing resistance to STb-induced damage.

## 1. Introduction

Heat-stable enterotoxin (ST), a toxin secreted by enterotoxigenic *Escherichia coli* (ETEC), has garnered significant attention in both livestock production and clinical research due to its severe diarrheagenic effects on both humans and animals [1]. Based on biological and pathogenic properties, ST is classified into STa and STb [2]. Furthermore, STa can be further categorized based on the ETEC source: STp, detected in cattle or pig strains, and STh, found in human strains. In contrast, STb can bind to both human and animal intestinal epithelium, exhibiting a more potent destructive capacity [3].

The intestinal epithelium serves as a key interface between the body’s internal and external environments, with its function regulated by various epithelial cell types, including absorptive cells, goblet cells, and Paneth cells [4]. Absorptive cells are responsible for nutrient absorption from the lumen, acting as the primary executors of intestinal digestion and absorption [5]. Goblet cells produce biomolecules such as mucins, trefoil factor 3 (TFF3), and resistin-like molecule β (RELM-β), which are key components of the intestinal chemical barrier and play an essential role in resisting pathogen invasion [6]. Additionally, Paneth cells, positioned in the crypts, secrete lysozyme to maintain the crypt’s environment and regulate intestinal stem cell (ISC) function by secreting niche factors [7]. All intestinal epithelial cells are derived from ISCs, which are organized along the crypt-villous axis, ensuring precise cell placement. Effective stem cell turnover requires a stable internal environment. Growing evidence indicates that intestinal stressors (e.g., deoxynivalenol [DON] and sodium dodecyl sulfate [SDS]) disrupt the redox balance by inhibiting the Kelch-like ECH-associated protein 1/Nuclear factor-erythroid 2- related factor 2 (Keap1/Nrf2) signaling pathway, thereby impairing the commitment lineage of ISCs [8,9]. As such, developing bioactive compounds targeting Nrf2 signaling represents a promising strategy for protecting gut health.

Iturin A, a bioactive peptide naturally synthesized by humans and animals’ intestinal probiotics, such as *Lactobacillus johnsonii*, *Bacillus subtilis* and *Bacillus amyloliquefaciens*, consists of a seven-amino-acid ring linked to a long chain of beta-amino fatty acids [10,11]. Evidence suggests that iturin A disrupts bacterial structures via its amino acid branch chains, thereby inducing death of the pathogenic bacteria [12]. Zhao et al. [12] demonstrated that oral administration of iturins to mice inhibited the colonization of intestinal Firmicutes and Proteobacteria while significantly enhancing the abundance of beneficial probiotics, such as Bifidobacterium. This suggests that iturins promote intestinal health through modulation of the microbiota. However, the potential of iturin A to modulate Keap1/Nrf2 signaling and mitigate STb-induced damage to intestinal stem cells remains unexplored.

This study evaluated the protective effects of iturin A on intestinal health in STb-challenged mice and investigated its underlying mechanisms. Our findings demonstrate that iturin A effectively modulates the Keap1/Nrf2 signaling pathway, restores the redox balance, and promotes the differentiation of ISCs into protective epithelial cells, thereby mitigating STb-induced damage. Taken together, these results suggest that iturin A could serve as a novel probiotic agent to regulate ISC function, offering a theoretical foundation for its application in practical settings.

## 2. Materials and Methods

### 2.1. Laboratory Equipment

A Ti2-U microscope (Nikon, Tokyo, Japan) was used to capture representative images of immunofluorescence (IHC) and Hematoxylin-Eosin (H&E) staining; a fluor chem m imaging system (Protein Simple, San Jose, CA, USA) was used to visualize Western blotting (WB) results; a full-temperature shaking incubator (Shanghai Zhichu Instrument Co., Ltd., Shanghai, China), ultrasonic cell breaker (Guangzhou Banghong Medical Equipment Co., Ltd., Guangzhou, China), and PCR instrument (TAKARA, Osaka, Japan) were used to carry out STb protein expression purification experiments.

### 2.2. In Vivo Assay

Seventy-two healthy, 4-week-old male C57BL/6 weaned mice with similar body weights were randomly divided into six groups (12 mice per group, 1 mouse per replicate): Control (CON) group (PBS), iturin A (IA) group (320 mg/kg BW iturin A, produced by Guangzhou Bai Shi Tai Biotechnology Co., Ltd., Guangzhou, China), STb-Rosetta group (STb-R) (2 × 10^11^ CFU bacterial solution), STb group (4 mg/kg BW STb), iturin A + STb-R group (320 mg/kg BW iturin A + 2 × 10^11^ CFU solution), and iturin A + STb group (320 mg/kg BW iturin A + 4 mg/kg BW STb). The STb-R group served as a positive control for the STb group, where mice were treated with a lysis solution of *E. coli* containing the STb-expressing plasmid. The mice were housed under 25 °C, 60% relative humidity and a regular 12 h:12 h light: dark cycle and kept free to eat and drink. After 7 days of continuous intragastric administration, mice were euthanized using CO_2_ inhalation, and intestinal tissues were collected. All the experiments were approved by the Animal Ethics Committee of South China Agricultural University (2023F356, Guangzhou, China).

### 2.3. Antibodies

Antibodies for WB and IHC included those against Claudin1 (#bs-1428R, Bioss, Beijing, China), Cleaved-caspase3 (C-Caspase3, #9664, Cell Signaling Technology, Boston, MA, USA), Cleaved Poly ADP-ribose polymerase (C-PARP, #WL01932, Wanleibio, Shenyang, China), Glutathione Peroxidase 4 (GPX4, #R381958, Zen BioScience, Chengdu, China), Keap1 (#R26935, Zen BioScience, Chengdu, China), Keratin20 (KRT20, #13063, Cell Signaling Technology, Boston, MA, USA), Lysozyme (LYZ, #A0099, Dako, Copenhagen, Denmark), Mucin2 (MUC2, #A14659, ABclonal, Wuhan, China), Occludin (#502601, Zen BioScience, Chengdu, China), Proliferating Cell Nuclear Antigen (PCNA, #2586, Cell Signaling Technology, Boston, MA, USA), p-Nrf2 (#381559, Zen BioScience, Chengdu, China), SOX9 (#380995, Zen BioScience, Chengdu, China), Wheat germ agglutinin (WGA, #L9640, Sigma, St Louis, MO, USA), and β-actin (#sc-81178, Santa Cruz, Dallas, TX, USA), as well as anti-rabbit IgG (#511203, Zen BioScience, Chengdu, China) and anti-mouse IgG (#511103, Zen BioScience, Chengdu, China) secondary antibodies.

### 2.4. Recombinant Expression and Purification of STb

As described in previous studies, the STb coding sequence was inserted into the pET-32a plasmid and expressed using the *E. coli* prokaryotic system. The protein was then purified using a commercial His-tagged protein purification kit (Beyotime Biotech, Shanghai, China) and a 5 K ultrafiltration cube.

### 2.5. Measurement of Oxidative-Antioxidant Parameters

For sample collection, mouse eyeballs were excised to extract venous blood, which was subsequently centrifuged at 3000 rpm for 10 min to obtain serum. Additionally, jejunal mucosa samples were homogenized, and the supernatant was isolated after centrifugation at 12,000 rpm for 10 min. ROS (XYM9437001, X-Y Biotechnology, Shanghai, China), T-AOC (JL-T1386, Jonlnbio, Shanghai, China), SOD (JL12237), and GSH-PX (JL49904) levels in the jejunal mucosa homogenate supernatant or serum were detected using kits.

### 2.6. Paraffin Blocks Preparation

Fresh intestinal tissue was fixed in 4% paraformaldehyde for 24 h. The tissue was then dehydrated through a graded alcohol series, cleared with xylene, and embedded in paraffin, where it was cooled to form wax blocks. Finally, the blocks were sectioned into 5 μm slices, collected on adhesive slides, and dried at 37 °C.

### 2.7. Hematoxylin-Eosin Staining

Paraffin sections were stained sequentially with hematoxylin and eosin following the kit instructions. The height of the villi and the depth of the crypts were measured using ImageJ software (version 1.8.0 112, National Institutes of Health, Bethesda, MD, USA).

### 2.8. Immunohistochemical Staining

For IHC analysis, paraffin sections were first cleared in xylene and then subjected to antigen retrieval using sodium citrate solution. The sections were subsequently incubated with bovine serum albumin to block non-specific binding, followed by incubation with primary antibodies, cy3/FITC fluorescent labeling, and 4′,6-di-amidino-2-phenylindole (DAPI) staining. IHC staining images were captured using an inverted fluorescence microscope (Ti2-U, Nikon, Tokyo, Japan), and the fluorescence signal intensity was measured by ImageJ software (version 1.8.0 112, National Institutes of Health, Bethesda, MD, USA). For testing, the jejunum from the six mice closest to the mean weight in each group was selected. The mean value of the control group was set to 1 (STb-R and STb groups vs. CON, IA, IA + STb-R and IA + STb groups).

### 2.9. Real-Time qPCR

Following established protocols [13], RNA extracted from jejunal tissue was reverse-transcribed into cDNA templates. These were then combined with synergetic binding reagent (SYBR) mix (AG11701, ACCURATE BIOTECHNOLOGY (HUNAN) Co., Ltd., Changsha, China) and primers (primer sequences are shown in Supplementary Materials Table S1), and computed tomography (CT) values were obtained using the CFX96 Touch Deep Well Real-Time PCR Detection System. Gene abundance was calculated using the 2^−ΔΔCt^ method. For testing, the jejunal mucosa from the six mice closest to the mean weight in each group was selected. The mean value of the control group was set to 1 (STb-R and STb groups vs. CON, IA, IA + STb-R and IA + STb groups).

### 2.10. Western Blotting

For protein analysis, jejunal mucosa was homogenized in RIPA Lysis Buffer, and all protein samples were normalized to 1000 ng/mL. The protein samples and markers (DB245-10, MIKX Co., Ltd., Shenzhen, China) were then separated by sodium dodecyl sulfate-polyacrylamide gel electrophoresis (SDS-PAGE), transferred onto polyvinylidene fluoride (PVDF) membranes, and blocked with NcmBlot blocking buffer (P30500, New Cell & Molecular Biotech Co., Ltd., Suzhou, China). The membranes were incubated with primary and secondary antibodies. Enhanced chemiluminescence signals were detected using a FluorChem M apparatus (Protein Simple, Inc., Santa Clara, CA, USA), and the band densities were quantified with ImageJ software (version 1.8.0 112, National Institutes of Health, Bethesda, MD, USA). For testing, the jejunal mucosa from the six mice closest to the mean weight in each group was selected. The mean value of the control group was set to 1 (STb-R and STb groups vs. CON, IA, IA + STb-R and IA + STb groups).

### 2.11. Data Analysis

Data were analyzed using SPSS version 19.0 (SPSS Inc., Chicago, IL, USA) and are presented as mean ± SEM. Differences between the six groups were assessed using the least significant difference (LSD) multiple-range test following one-way ANOVA. Statistical significance was defined as *p*-values < 0.05, with different letters in the same column indicating significant differences. A statistical tendency was noted for 0.05 ≤ *p*-values < 0.10.

## 3. Results

### 3.1. Iturin A Attenuates STb Damage to Intestinal Epithelial Structures in Mice

**Figure 1:**
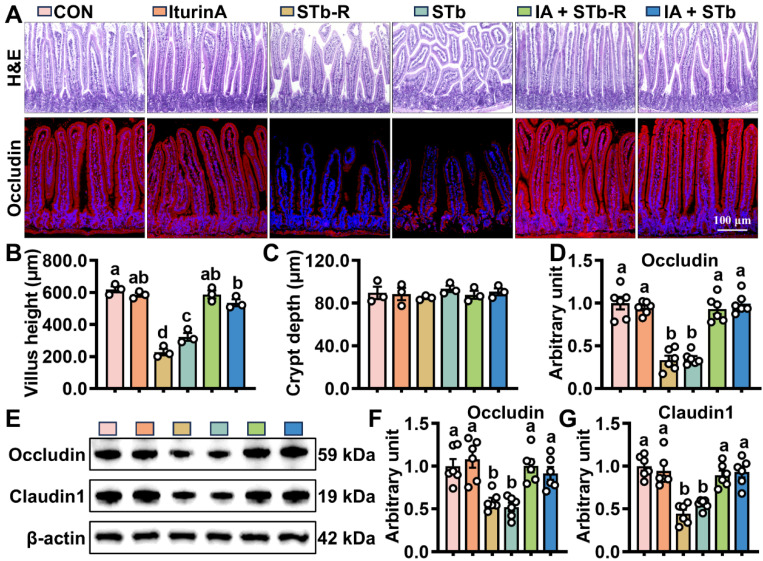
Iturin A accelerates jejunal mucosa repair in STb-exposed mice. (**A**–**C**) H&E staining of jejunal mucosa; (**A**,**D**) IHC staining of Occludin proteins in the jejunum (100× magnification) (n = 6, each white circle represents a sample repeat); (**E**–**G**) WB analysis of Occludin and Claudin1 expression in the jejunum (n = 6, each white circle represents a sample repeat). Data are presented as the mean ± SEM. Different lowercase letters indicate significant differences between the compared groups (*p* < 0.05).

To assess whether iturin A alleviates STb-induced damage, the bodyweight of each group of mice was statistically analyzed. The data showed that STb-R/STb treatment significantly impeded the growth of mice compared to CON (Figure S1). As shown in Figure 1, significant reductions in villus height and extensive structural damage were observed in the jejunum of STb/STb-R-exposed mice, with no substantial changes in crypt depth (Figure 1A–C). Additionally, STb/STb-R exposure suppressed the fluorescent signal of Occludin (Figure 1A,D), and significantly decreased the protein expression of Occludin and Claudin1 (Figure 1E–G), compromising the epithelial barrier function. In contrast, in the iturin A-treated group, all the crypt-villus structure, fluorescence signals of tight junction proteins, and body weight were restored to levels comparable to the CON (Figure 1A–G and Figure S1). These results suggest that iturin A supplementation facilitates the reconstruction of the epithelial structure and enhances barrier function following STb/STb-R insult.

### 3.2. Iturin A Enhances the Activity of Mouse ISCs After STb Invasion

**Figure 2:**
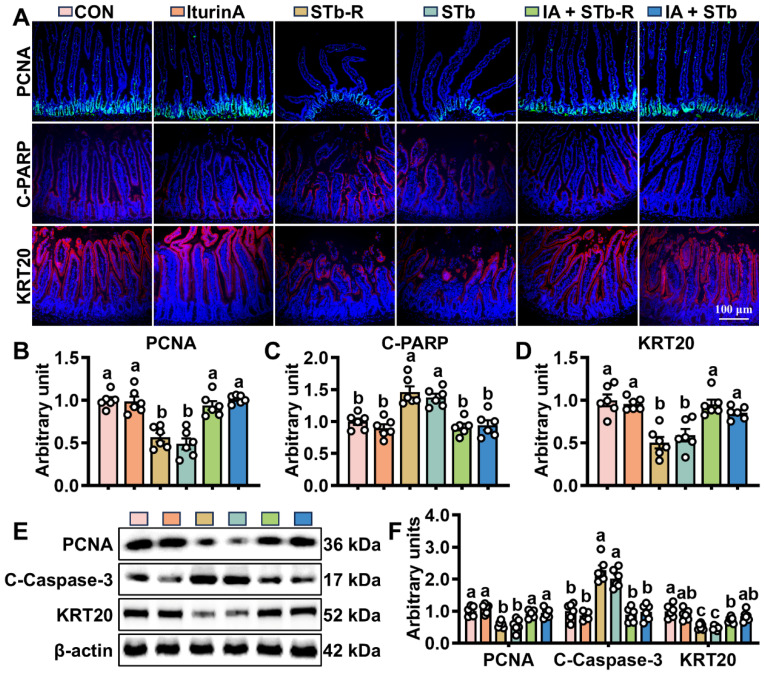
Iturin A stimulates ISC proliferation in STb-challenged mice. (**A**–**D**) IHC staining of PCNA, Cleaved PARP, and KRT20 proteins in the jejunum (100× magnification) (n = 6, each white circle represents a sample repeat); (**E**,**F**) WB analysis of PCNA, Cleaved Caspase-3, and KRT20 expression in the jejunum (n = 6, each white circle represents a sample repeat). Data are presented as the mean ± SEM. Different lowercase letters indicate significant differences between the compared groups (*p* < 0.05).

**Figure 3:**
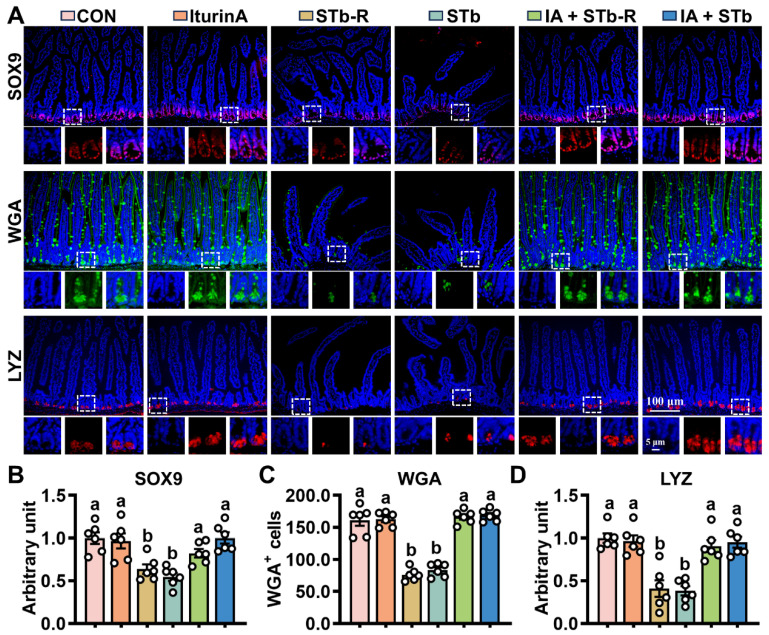
Iturin A promotes the differentiation of STb-exposed ISCs into goblet and Paneth cells. (**A**–**D**) IHC staining of SOX9, WGA, and LYZ proteins in the jejunum (200× and 400× magnification) (n = 6, each white circle represents a sample repeat); Data are presented as the mean ± SEM. Different lowercase letters indicate significant differences between the compared groups (*p* < 0.05).

ISCs regulate epithelial cell turnover by modulating proliferation and differentiation. Changes in stem cell viability were assessed by WB and IHC, revealing that STb/STb-R exposure downregulated PCNA and KRT20 expression while increasing the levels of Cleaved-PARP and Cleaved-caspase3 (Figure 2A–F). Furthermore, STb/STb-R exposure disrupted ISC differentiation into secretory cells, significantly reducing the proportion of SOX9+, MUC2+, and LYZ+ cells (Figure 3A–D). However, iturin A supplementation upregulated PCNA and KRT20 expression and increased the number of SOX9+, MUC2+, and LYZ+ cells (Figure 2 and Figure 3). Consistently, iturin A reversed changes in Spdef (goblet cell marker) and Defensin24 (Defa24, Paneth cell marker) gene abundance with STb-R/STb exposure (Figure S2). These results indicate that iturin A promotes ISC proliferation and restores normal differentiation patterns in response to STb/STb-R-induced damage.

### 3.3. Iturin A Elevates Intestinal Antioxidant Capacity in Mice After STb Invasion

**Figure 4:**
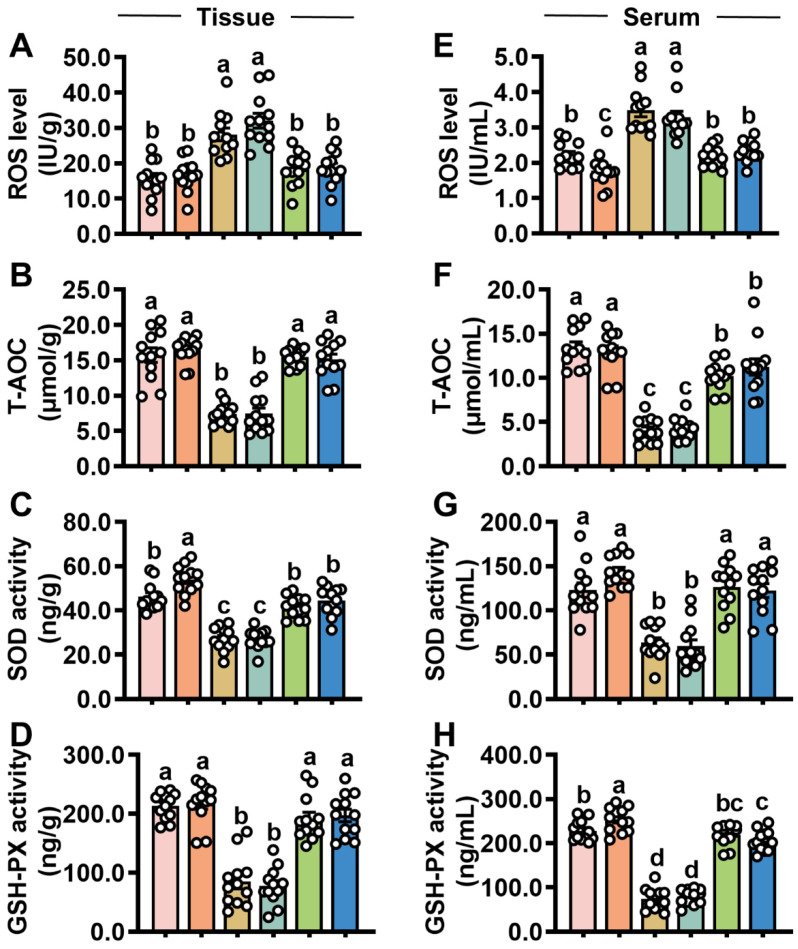
Iturin A prevents STb from disrupting redox homeostasis in the mouse jejunal mucosa. (**A**–**D**) The levels of ROS, T-AOC, SOD, and GSH-PX in the jejunal mucosa; (**E**–**H**) The levels of ROS, T-AOC, SOD, and GSH-PX in serum (n = 12, each white circle represents a sample repeat). Data are presented as the mean ± SEM. Different lowercase letters indicate significant differences between the compared groups (*p* < 0.05).

A stable redox environment is essential for the proper turnover of the intestinal epithelium. In the STb/STb-R group, T-AOC, SOD and GSH-PX in jejunal mucosa (Figure 4B–D) and serum (Figure 4F–H) were significantly reduced, while reactive oxygen species (ROS) levels (Figure 4A,E) were markedly elevated. These results suggest that STb/STb-R disrupts the redox balance in mice. However, iturin A treatment reversed these changes, restoring antioxidant capacity in STb/STb-R-exposed mice (Figure 4A–H).

### 3.4. Iturin A Promotes Intestinal Keap1/Nrf2 Signaling in Mice Under STb Exposure

**Figure 5:**
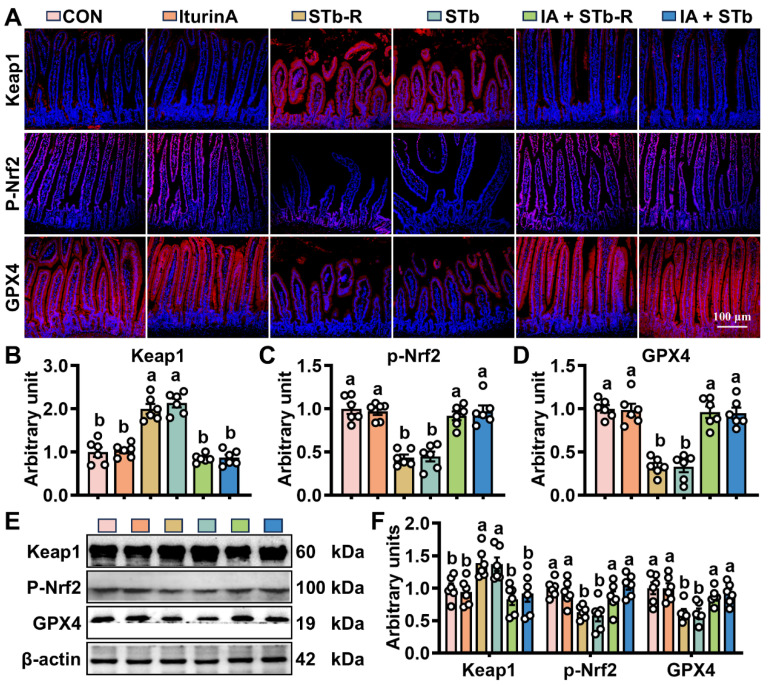
Iturin A promotes intestinal Keap1/Nrf2 signaling in mice exposed to STb. (**A**–**D**) IHC staining of Keap1, p-Nrf2, and GPX4 proteins in the jejunum (100× magnification) (n = 6, each white circle represents a sample repeat); (**E**,**F**) The expression of Keap1, p-Nrf2, and GPX4 proteins in the jejunum (100× magnification) (n = 6, each white circle represents a sample repeat). Data are presented as the mean ± SEM. Different lowercase letters indicate significant differences between the compared groups (*p* < 0.05).

The Keap1/Nrf2 signaling pathway plays a pivotal role in regulating the body’s redox balance. The IHC of key proteins in this pathway showed that STb/STb-R treatment led to a significant increase in the Keap1 fluorescence signal, while p-Nrf2 and GPX4 fluorescence intensity were reduced (Figure 5A–D). Iturin A treatment notably inhibited Keap1 expression and restored p-Nrf2 levels. Consistent with this, WB analysis confirmed that iturin A effectively alleviated the inhibitory effect of STb/STb-R on Keap1/Nrf2 signaling, leading to an upregulation of the downstream antioxidant protein GPX4 (Figure 5E,F).

## 4. Discussion

The intestinal epithelium, consisting of tight junction proteins and various intestinal cells, serves as a physical barrier by tightly connecting the cells, allowing only nutrients to pass while blocking toxin molecules in the lumen [14]. However, sustained stress can inhibit the expression of tight junction proteins, leading to the collapse of the intestinal epithelial structure [15,16]. For instance, researchers have reported that injecting DON or subjecting mice to high-temperature stress for seven consecutive days resulted in reduced tight junction protein expression, villus atrophy, fragmentation, and decreased crypt depth [17,18]. In the present study, STb exposure significantly shortened the height of jejunal villi, while fluorescence signals for Occludin and Claudin1 were weakened. Interestingly, the damage caused by STb appeared to be limited to the villus region, with no structural changes in the crypts. This may be due to the reverse fluid flow in the crypts [19], which effectively prevented the invasion of STb. Recent studies have explored the use of biological substances, such as bacitracin and collagen peptides, to promote tissue repair by providing protein synthesis substrates [20,21,22]. Our findings also suggest that iturin A can effectively protect the intestinal epithelium of mice from STb-induced damage. However, further investigation is required to determine whether iturin A, an antimicrobial peptide, can mitigate injuries beyond bacterial toxins, such as those caused by mechanical stress or radiation.

The stable turnover of ISCs is critical for maintaining the biological function of the intestinal epithelium [23]. Toxic substances typically disrupt ISC mitosis, trigger cell death, and ultimately impair epithelial function [24]. In this study, STb exposure significantly reduced the number of PCNA+ cells and increased levels of Cleaved-PARP (a protein associated with apoptosis) in the jejunum. This may result from STb’s disruption of the chemical barrier, inhibiting the differentiation of ISCs into defensive epithelial cells (such as goblet and Paneth cells), thereby exacerbating the damage and leading to cell collapse. Additionally, studies have reported that STb lowers intestinal pH, contributing to an unfavorable environment that inhibits ISC activity [2]. Further, STb impaired the antioxidant system, causing the accumulation of ROS, further confirming that STb compromises ISC function by disrupting the intestinal microenvironment. Numerous studies have shown that bioactive peptides from animal and plant sources, such as transferrin peptide, wasp venom peptide, pig intestinal mucosa enzymatic hydrolysis peptides, and plant complex peptides, can protect intestinal cells from oxidative damage [25,26,27,28]. Consistently, our data demonstrate that iturin A enhances antioxidant capacity in STb-challenged mice and restores the normal differentiation pathway of ISCs, highlighting the significant potential of iturin A in regulating redox balance and maintaining intestinal health. Notably, iturin A intervention improved redox markers in the serum of STb-challenged mice, indicating its ability to enter the bloodstream. And this underscores the importance of evaluating the biosafety of iturin A through multi-organ indicators prior to clinical application.

The stable conduction of the Keap1/Nrf2 signaling pathway is essential for maintaining the homeostasis of the internal environment, as it is the primary regulator of the redox balance [29,30,31]. Numerous studies have demonstrated that ETEC infection disrupts Keap1/Nrf2 signaling, leading to intestinal oxidative stress [32,33]. As expected, in this study, STb, derived from ETEC, upregulated Keap1 protein expression, downregulated Nrf2 levels, and inhibited the production of the downstream antioxidant enzyme GPX4. Notably, Keap1 does more than bind to Nrf2; it also degrades SOX9, a protein critical for the maintenance of secretory progenitor cells [34,35]. This provides compelling evidence for the loss of Paneth and goblet cells following STb exposure. The role of Keap1/Nrf2 signaling in controlling redox balance has inspired researchers to explore this pathway for developing intestinal protective agents. For instance, peptides such as soybean antioxidant peptide Iunasin and β-lactoglobulin heptapeptide have been shown to increase Nrf2 levels [36,37]. Similarly, our study found that iturin A can inhibit Keap1 expression and promote the accumulation of Nrf2. The mechanism by which iturin A regulates Keap1/Nrf2 signaling may be similar to that of polyunsaturated fatty acids, which modify key cysteine residues on Keap1 through their α- and β-unsaturated bonds [11,38]. This modification causes conformational changes in the Keap1 dimer, ultimately leading to the release of Nrf2. However, whether iturin A directly interacts with Keap1 in a similar manner to peptides like Ile-Cys-Arg-Asp (LCRD) and Leu-Cys-Gly-Glu-Cys (LCGEC) remains an area for further exploration [39].

## 5. Conclusions

This study is the first to demonstrate that iturin A enhances the activity of ISCs by promoting Keap1/Nrf2 signaling, thereby mitigating the damage caused by STb. These findings lay a solid foundation for the potential application of iturin A as a novel therapeutic agent for protecting intestinal health and motivate us to continue to optimize the purification process of iturin A to meet the criteria for human clinical drug applications.

## Data Availability

The data that support the findings of this study are available from the corresponding author upon reasonable request.

## Supplementary Materials

The following supporting information can be downloaded at: https://www.mdpi.com/article/10.3390/antiox14040478/s1, Figure S1: Iturin A promotes growth in STb-R/STb-exposed mice.; Figure S2: Iturin A increases gene abundance of *Spdef* and *Defa24* in the jejunal mucosa of STb-challenged mice; Table S1: Primers used in this study.
