# Supplementary material for: Iturin A Potentiates Differentiation of Intestinal Epithelial Defense Cells by Modulating Keap1/Nrf2 Signaling to Mitigate Oxidative Damage Induced by Heat-Stable Enterotoxin B
Source: Antioxidants (Basel). 2025 Apr 16;14(4):478. doi: 10.3390/antiox14040478 (PMC12024041; doi:10.3390/antiox14040478)
Supplement: Supplementary file 1 [file antioxidants-14-00478-s001.zip › antioxidants-3546246-supplementary.pdf]

Supplementary information

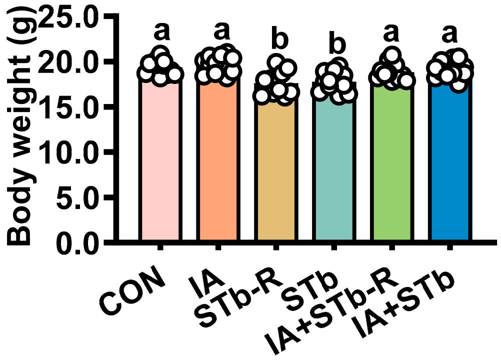

Figure S1 Iturin A promotes growth in STb-R/STb-exposed mice.

body weight (n = 12, each white circle represents a sample repeat). Data are presented as the mean ± SEM. Different lowercase letters indicate significant differences between the compared groups ($P < 0.05$).

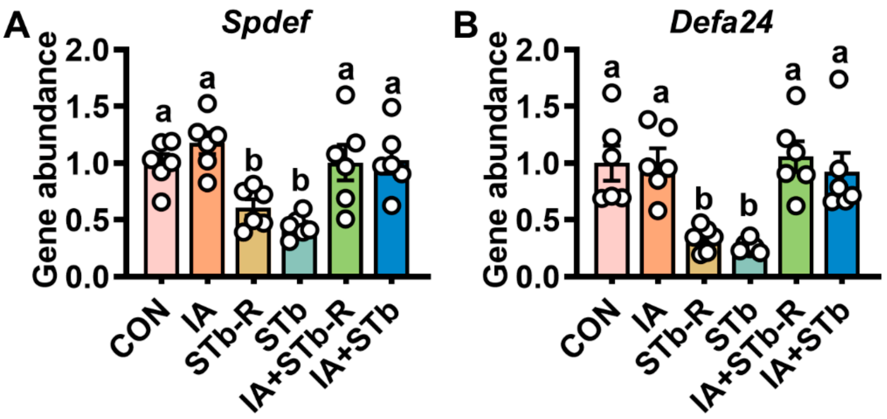

Figure S2 Iturin A increases gene abundance of *Spdef* and *Defa24* in the jejunal mucosa of STb-challenged mice.

(A-B) The gene abundance of *Spdef* and *Defa24* (n = 6, each white circle represents a sample repeat). Data are presented as the mean ± SEM. Different lowercase letters indicate significant differences between the compared groups ($P < 0.05$).

Supplementary Table S1 Primers used in this study

Gene	ID	Forward primer (5′-3′)	Reverse primer (5′-3′)
Spdef	30051	TTGGATGAGCACTCGCTAGA	AAAAGCCACTTCTGCACGTT
Defa24	503491	TGTAGAGCAAGAGGCTGCAA	CAGCATCAGTGGCCTCAGTA
GAPDH	14433	GAAGGTGAAGGTCGGAGTC	GAAGATGGTGATGGGATTTC
